# Neonatal SSRI Exposure Programs a Hypermetabolic State in Adult Mice

**DOI:** 10.1155/2012/431574

**Published:** 2012-04-10

**Authors:** Gary J. Kummet, Sarah E. Haskell, Gregory M. Hermann, Charles Ni, Kenneth A. Volk, Areej K. Younes, Alise K. Miller, Robert D. Roghair

**Affiliations:** Department of Pediatrics, Carver College of Medicine, University of Iowa Carver College of Medicine, Iowa City, IA 52242, USA

## Abstract

*Background*. Selective serotonin reuptake inhibitor (SSRI) therapy complicates up to 10% of pregnancies. During therapy, SSRIs exert pleiotropic antidepressant, anorexigenic, and neurotrophic effects. Intrauterine SSRI exposure has been modeled by neonatal administration to developmentally immature rodents, and it has paradoxically elicited features of adult depression. We hypothesized neonatal SSRI exposure likewise programs a rebound hypermetabolic state in adult mice. *Methods*. C57BL/6 pups were randomized to saline or sertraline (5 mg/kg/d) from P1–P14. Because estrogen increases tryptophan hydroxylase 2 (TPH2) expression, a subset of female mice underwent sham surgery or bilateral ovariectomy (OVX). Metabolic rate was determined by indirect calorimetry. *Results*. In both male and female mice, neonatal SSRI exposure increased adult caloric intake and metabolic rate. SSRI-exposed female mice had significantly decreased adult weight with a relative increase in brain weight and melatonin excretion, independent of ovarian status. Cerebral cortex TPH2 expression was increased in SSRI-exposed male mice but decreased in OVX SSRI-exposed female mice. *Conclusions*. SSRI exposure during a critical neurodevelopmental window increases adult caloric intake and metabolic rate. Ovarian status modulated central TPH2 expression, but not adult energy balance, suggesting programmed neural connectivity or enhanced melatonin production may play a more important role in the post-SSRI hypermetabolic syndrome.

## 1. Introduction

The prevalence of depression during pregnancy now exceeds 18%, and more than 13% of pregnancies were complicated by antidepressant therapy in 2003, twice as many as in 1999 [1, 2]. The majority of this increase has come from a heightened utilization of selective serotonin reuptake inhibitors (SSRIs) [2–6]. Among the SSRIs, sertraline remains the most commonly prescribed antidepressant in America [2].

Recent studies have demonstrated detrimental effects of *in utero* SSRI exposure, including fetal growth restriction, neonatal abstinence syndrome, and persistent pulmonary hypertension [5, 7]. The effects of third trimester exposure are often felt to be transient in nature. However, Oberlander and colleagues have demonstrated changes in hypothalamic, cardiovascular, and nociceptive regulation up to 4 months following delivery, and SSRI exposure has now been associated with increased autism risk [8–13].

Murine models of human neurodevelopment are well established, with the first fourteen postnatal days approximating the third trimester of human development [14]. Exposure of male rats to the SSRI citalopram at the later end of this critical window (P8–P21) decreases dorsal raphe tryptophan hydroxylase (TPH) immunoreactivity and elicits behavioral changes consistent with depression [15]. Further studies on that model have identified SSRI-induced disruptions in neural networks and upregulation of the noradrenergic locus ceruleus system [16, 17]. When reported, outcomes have been blunted in female mice and rats [16, 17]; suggesting estrogen-stimulated TPH expression may provide partial protection [18].

We sought to expand the assessment of SSRI-programmed phenotypes and determine the sex-specific effects of neonatal exposure to sertraline, the most commonly prescribed antidepressant. Given the known anorexigenic and sympathoinhibitory effects of acute SSRI administration [19–21], we speculated alterations in energy balance may be a component of a potential “post-SSRI syndrome.” We hypothesized that SSRI-exposed male and ovariectomized female mice have decreased adult serotonergic tone that is manifest by increased feed intake and basal metabolic rate.

## 2. Methods

### 2.1. Animal Model

 Pregnant C57BL/6 mice (Jackson Laboratory, Bar Harbor, ME) were allowed natural delivery and within 12 hours of birth, each litter was adjusted to 6 pups, via culling or adding age-matched pups from other dams. While C57BL/6 dams readily accept cross-fostered pups [22, 23], a recent publication described alterations in adult outcomes when entire litters were cross-fostered at 48 h [23]. A vast majority of litters did not require any additional pups, and the confounding effects of cross-fostering were minimized by randomization. Pups were then randomized within each litter to either receive intraperitoneal saline (10 mL/kg/d) or sertraline (5 mg/kg/d) on days P1–14. This corresponds to a neurodevelopmental window similar to the third trimester in human gestation. The dose utilized was determined from the equation: murine dose = maternal oral dose/maternal body surface area × murine body surface area × oral bioavailability × placental transfer ratio. Utilizing the equation of Meeh for murine body surface area [9.8 × (weight in g)^2/3^] [24], the average weight of mice at 14 d (7 g), the equation of Du Bois for human body surface area [71.84 × (weight in kg)^0.425^ × (height in cm)^0.725^] [25], the average weight and height of women in the third trimester (80 kg, 162 cm), 70% oral bioavailability and 29% placental transfer [26], the equation simplifies to murine dose = maternal dose ∗ 0.00039. Thus, to replicate typical low-dose therapy of 100 mg/d, we utilized 0.039 mg/d or ~5.6 mg/kg/d. To verify the clinical relevance of this dosing regimen, plasma was collected 2 h or 12 h after the final dose of sertraline. Prior to collection, pups were anesthetized with isoflurane (1%), the liver was excised and 600–750 microliter of blood was collected. Plasma was stored at −20°C prior to analysis by gas chromatography at NMS Labs (Willow Grove, PA).

To further assess the protective effect of ovarian function, a subset of 5- to 6-month-old female mice underwent bilateral ovariectomy (OVX) versus sham surgery (ovary visualization without resection) via paravertebral approach under isoflurane anesthesia. Analgesia was provided with flunixin meglumine (2.5 mg/kg once or twice daily), as well as 0.5% bupivacaine along the incisions. A minimum of one month of surgical recovery was provided prior to phenotyping. All surgeries and protocols were in accordance with NIH guidelines and were approved by the Institutional Animal Care and Use Committee at the University of Iowa. All investigations were designed to reduce the numbers of animals required and procedures were modified to lessen or eliminate pain and distress.

### 2.2. *In Vivo* Phenotypes

Feed consumption and weight were measured over a 14-day interval while mice received *ad lib* standard rodent chow (4 kcal/g, 6% of energy as fat; 7013; Harlan Teklad, Madison, WI). Basal metabolic rate was then assessed via indirect calorimetry in an airtight thermoneutral chamber. Oxygen consumption (VO2) was calculated while the mice were asleep, as previously described [27]. For male mice, physiologic studies (performed at 5–7 months) were followed by brain harvest under general anesthesia at 6–9 months. The female mice that underwent ovariectomy or sham surgery had delayed euthanasia (11-12 months). During this delay, analysis of the *in vivo* phenotype data led us to place them in a metabolic cage for determination of 24 h urinary melatonin excretion. Samples were stored at −80 degrees until analysis in duplicate for melatonin sulfate (ELISA kit RE54031, IBL Transatlantic). Samples with no detectible melatonin sulfate (lower limit of detection 1 ng/mL) were analyzed with a value of zero. There was one such sample present in each of the 4 groups. Following these studies, female mice were euthanized by organ harvest under general anesthesia, and tissue weights were obtained.

### 2.3. Tryptophan Hydroxylase Expression

The brain was quickly and bluntly segmented and stored in RNAlater until purification with RNeasy kits (Qiagen, Valencia, CA). Initial coronal sectioning removed the olfactory bulbs anteriorly, as well as the cerebellum and medulla posteriorly. The remaining brain was then sectioned both superiorly and laterally to obtain a sample labeled “cortex” including both the cerebral cortex and pineal gland. The remaining segment, labeled “midbrain” included the dorsal raphe nucleus as well as the diencephalon (thalamus and hypothalamus). Finer dissection was not completed to avoid loss of message due to either passage of time or indiscriminate removal of grossly indistinct regions. RNA was quantitated using a NanoDrop ND-1000 spectrophotometer (Labtech International, East Sussex, UK). Reverse-transcription reactions were performed on 0.5 *μ*g total RNA with the addition of oligo dT, dNTPs, DTT, RNasin, and Superscript III reverse transcriptase (Invitrogen). Quantitative real-time RT-PCR (qPCR) utilized the TaqMan reagent and instrumentation systems (Applied Biosystems, Foster City, CA). Taqman gene expression assay primer/probe sets for mouse Tph1 (assay ID = Mm00493794_m1; context sequence = CCGACCACCCTGGCTTCAAAGACAA), Tph2 (assay ID = Mm00557715_m1; context sequence = TAGACTATTCCAGGAAAAACATGTC), and GAPDH were purchased from Applied Biosystems (Foster City, CA). Since the reaction efficiencies for the 3 assays are matched by design, we used the ΔΔCT method for quantitation.

### 2.4. Data Analysis

All values other than TPH expression data are presented as mean ± SEM. TPH expression is presented as % control = 100 × 2^(−ΔΔCT)^ with corresponding error bars equal to 100 × 2^(−ΔΔCT ± pooled SE)^. Statistical comparisons were made using two-way ANOVA and the Holm-Sidak method for multiple pair-wise comparisons. When ANOVA identified a significant interaction between SSRI exposure and ovarian status, pairwise comparisons were made by the Holm-Sidak method. Postmortem tissue weights were compared by Student's *t*-test. A value of *P* < 0.05 was considered significant. All analyses were performed using SigmaPlot 12 (Systat Software Inc.).

## 3. Results

### 3.1. Acute Sertraline Administration Decreases the Growth of Neonatal Mice and Models Intrauterine Human Exposure

**Figure 1:**
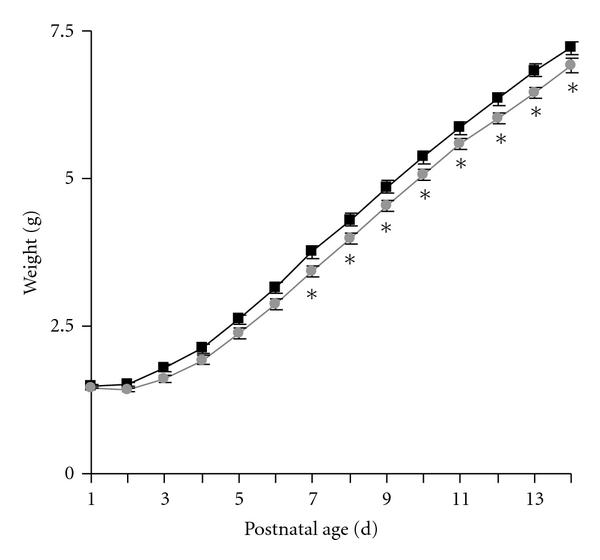
Neonatal SSRI exposure acutely decreased pup growth. Pups were weighed immediately prior to daily administration of 10 mL/kg saline (white symbols) or 5 mg/kg sertraline (gray symbols) from P1 to P14. *N* = 34 control and 40 SSRI-exposed pups. *P < 0.05 by two way repeated measures ANOVA with Holm-Sidak method for multiple pair-wise comparisons.

Pup weights at the initiation of sertraline exposure (P1) were similar (control 1.49 ± 0.02 g, SSRI-exposed 1.46 ± 0.02 g). Throughout the final 9 days of injections (P6–P14), sertraline-exposed mice had mild but statistically significant growth restriction (Figure 1, overall ANOVA *P* = 0.032, difference of means 0.26 g). On the final day of exposure (14 d), plasma levels 2 h after injection were 71.8 ± 1.3 ng/mL (*N* = 6) and 12 h after injection were 13.1 ± 0.6 ng/mL (*N* = 7). Based on these levels, there was a half-life 4.1 h, estimated peak concentration 101 ng/mL, and estimated trough concentration of 1.7 ng/mL. Our projected peak concentration approximates that seen in pregnancy (99 ng/mL based on 150 mg/d dosing) [28], and our projected trough approximates umbilical cord levels (4.9 ng/mL) [26]. The mean plasma level in mice (24.8 ng/mL) closely approximates mean human levels measured in the general population (20.4 ng/mL) and in pregnant women (also 20.4 ng/mL) [26, 29].

### 3.2. Neonatal SSRI Exposure Induces a Hypermetabolic State

**Figure 2:**
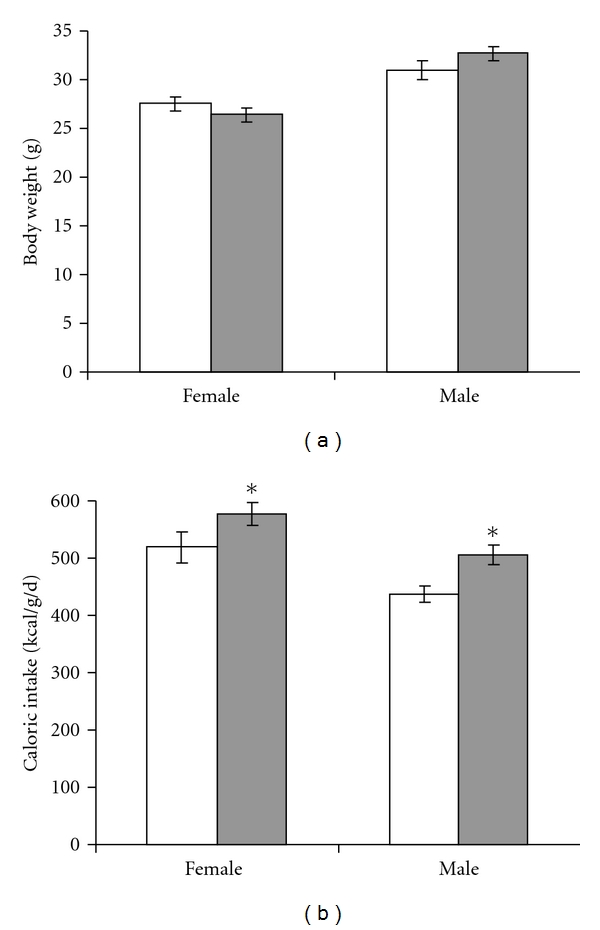
Neonatal SSRI exposure increases adult caloric intake. Intake of standard rodent chow was recorded over a 2 week interval and compared between control mice that received 10 mL/kg/d saline (white bars) and SSRI-exposed mice that received 5 mg/kg/d sertraline (gray bars) from P1 to P14. *N* = 27 control female (16 litters), 24 SSRI-exposed female (14 litters), 14 control male (9 litters), 12 SSRI-exposed male (10 litters); *P = 0.004 versus control by ANOVA.

**Figure 3:**
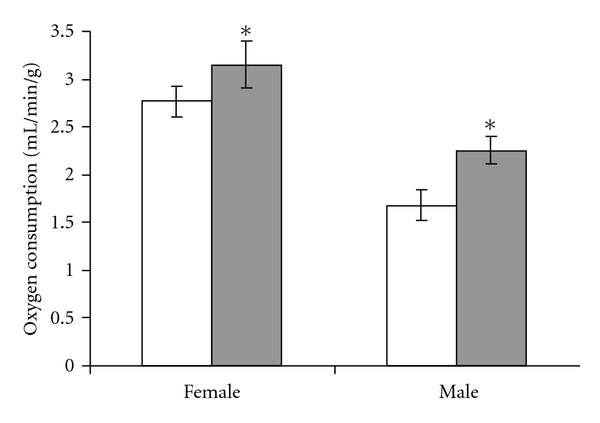
Neonatal SSRI exposure increases adult basal metabolic rates. Control (white bars) and SSRI-exposed mice (gray bars) were placed in a thermoneutral environment and oxygen consumption rates were measured during sleep (nadir in oxygen consumption). *N* = 27 control female (17 litters), 24 SSRI-exposed female (15 litters), 12 control male (9 litters), 13 SSRI-exposed male (10 litters); *P = 0.033 versus control by ANOVA.

Body weight had normalized at the beginning of adult phenotype assessment at 5–7 months (Figure 2(a)). At this point, mice were separated into individual cages to quantify feed intake. Sertraline exposure increased adult male and female caloric intake (Figure 2(b), *P* = 0.004). This increased caloric intake was matched by an increased basal metabolic rate (resting oxygen consumption) in both male and female SSRI-exposed mice (Figure 3, *P* = 0.033). Upon necropsy, SSRI-exposed female and male mice had decreased body weight (Table 1, *P* = 0.021 and *P* = 0.048, resp.) with increased relative brain weight seen in females (*P* = 0.017) but not males (*P* = 0.56). Additional organ weights were not obtained from male mice. Among female mice, there were no significant alterations in the absolute or relative weights of the liver (*P* = 0.07), intraabdominal white adipose tissue (*P* = 0.09), or interscapular brown adipose tissue (*P* = 0.85), although body composition tended to be leaner in the growth-restricted SSRI-exposed mice (Table 1).

### 3.3. Neonatal Sertraline Exposure Increases Adult Tryptophan Hydroxylase Expression and Melatonin Excretion

**Figure 4:**
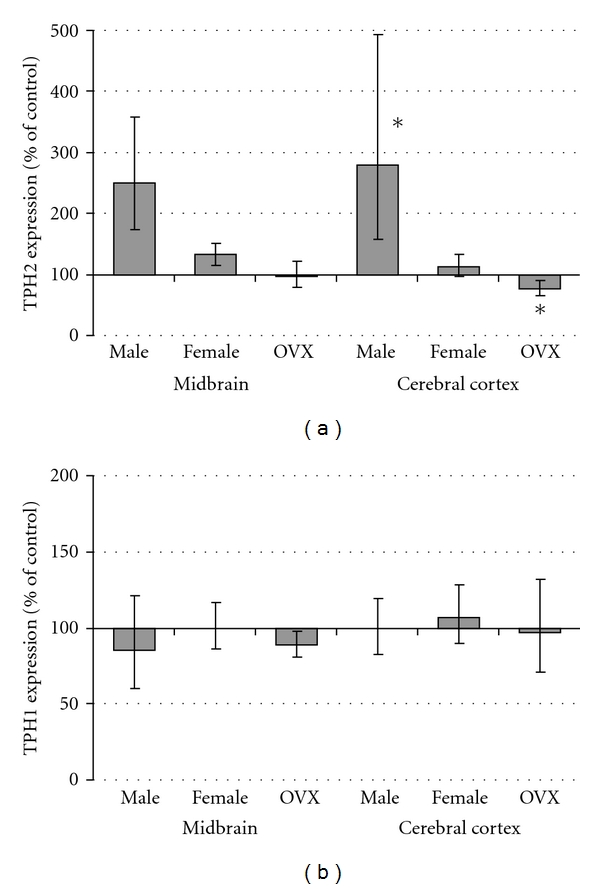
Cerebral cortex expression of TPH2 mRNA is increased in SSRI-exposed male mice. PCR was performed on midbrain and cerebral cortex homogenates obtained from control and SSRI-exposed mice (gray bars). Expression of the two tryptophan hydroxylase isoforms (TPH2 and TPH1) was normalized by GAPDH and ΔΔCT values were calculated to determine relative mRNA abundance. *N* = 5–10 mice from 5–8 litters per group; *P < 0.05 versus control by ANOVA with the Holm-Sidak method for multiple comparisons.

**Figure 5:**
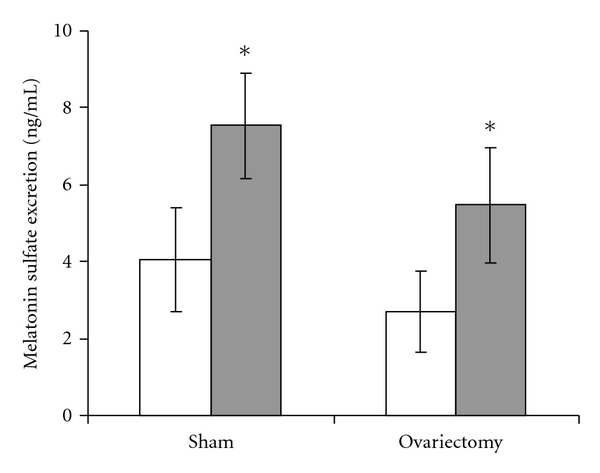
Urinary excretion of the major melatonin metabolite is increased by neonatal SSRI exposure independent of ovarian status. Mice were placed in metabolic cages for 24 hours and urinary excretion of melatonin sulfate was quantified by ELISA for samples from control (white bars) and SSRI-exposed mice (gray bars) that had undergone sham surgery or bilateral ovariectomy at least one month prior to collection. *N* = 5–9 mice from 5–7 litters per group; *P = 0.034 versus control by ANOVA, *P* = 0.23 for ovariectomy versus sham surgery.

In order to assess central serotonergic tone, regional and isoform-specific TPH expression was determined. By qPCR (Figure 4(a)), SSRI-exposed male mice had increased expression of the major TPH isoform (TPH2) within the cerebral cortex (2.8-fold increase, *P* = 0.048) and midbrain (2.5-fold increase, *P* = 0.11). Among female mice, there was an interaction between SSRI exposure and OVX (*P* = 0.017), with significantly decreased TPH2 expression in OVX SSRI-exposed mice cortex (*P* = 0.027). Brain TPH1 mRNA expression was not altered by SSRI exposure or ovarian status (Figure 4(b)). Overall, TPH2 mRNA expression was enriched 143-fold in the midbrain (ΔCT: 4.2 ± 0.3) versus the cortex (ΔCT: 11.3 ± 0.2), while TPH1 mRNA expression was increased 2-fold in the cortex (ΔCT: 10.8 ± 0.1) over the midbrain (ΔCT: 11.9 ± 0.1). Because a vast majority of excreted serotonin metabolites originate from systemic rather central sources, we chose to measure urinary excretion of the stable melatonin metabolite, melatonin sulfate [30]. Unlike serotonin produced within the dorsal raphe, melatonin produced within the pineal gland readily enters the systemic circulation such that urinary excretion allows for noninvasive approximation of central production. Independent of ovarian status, neonatal SSRI exposure increased urinary melatonin sulfate excretion (Figure 5, *P* = 0.034).

## 4. Discussion

Over the past decade, a growing percentage of infants have been exposed to maternal antidepressant therapy without long-term outcome data. Animal models have been developed to fill this void. Advantages of murine models of human exposures include a shortened lifespan allowing life-course assessment and the availability of isogenic inbred strains. To capitalize on these advantages, a well-timed clinically relevant environmental exposure must be introduced during a critical window of developmental susceptibility. Intrauterine SSRI exposure induces cardiac malformations in mice that are reminiscent of the congenital cardiac defects seen with intrauterine SSRI exposure in humans [31–34]. However, the third trimester of human neurodevelopment is best modeled in neonatal mice and rats. In rats, neonatal SSRI exposure decreases synaptogenesis and elicits features consistent with depression [15–17]. This post-SSRI syndrome appeared to have a sexually dimorphic presentation with male mice affected more than females. Our well-powered investigations clarify and extend these studies in demonstrating an SSRI-programmed hypermetabolic state in both male and female mice. Our data further demonstrate that while ovarian function modulates serotonergic tone, females are not sheltered from the effects of neonatal SSRI exposure.

Our dosing regimen (5 mg/kg once daily) was designed to replicate intrauterine exposure to maternal doses of only 100 mg once daily, and it matches the lowest exposure used in analogous preclinical studies [35]. Based on studies showing dose-dependent growth inhibition by sertraline [35], it is possible higher exposures may elicit greater programming effects. Consistent with the results seen in rats, neonatal mice have faster sertraline elimination (half-life 4 h) than young women (half-life 32 h) [36, 37]. We utilized a typical once daily dosing regimen that led to slightly exaggerated peak and trough fluctuations. Further studies with a more frequent dosing interval are needed to determine the programming effects of the absolute exposure versus the pattern of exposure and withdrawal. Notably, intrauterine SSRI exposure-associated neonatal abstinence syndrome appears to increase the risk for persistent social-behavioral abnormalities [5, 38].

The increased feed intake we identified in SSRI exposed adult mice contrasts with the anorexigenic effect of acute SSRI therapy [39, 40]. This is consistent with the developmental origins “predictive adaptive responses” theory or the broader developmental biology principle of phenotypic plasticity [41]. Both fields agree that when normal physiologic processes are perturbed by environmental exposures, the organism adapts to restore homeostatic balance. However, if the exposure ceases after this window of developmental plasticity closes, the adaptive response may be fixed and exert maladaptive effects. The remainder of our investigations sought to unravel the developmental origins of this SSRI-induced adult hyperphagia.

Intriguingly, despite the increased caloric intake of SSRI-exposed mice, body weight was either maintained or decreased compared to saline-exposed controls. While the concomitant increase in metabolic rate seen in SSRI-exposed mice may be explanatory, further studies are necessary to define whether the increased feed intake is a primary alteration or a reaction to the increased energy utilization. The diminished weight gain seen in SSRI-exposed mice between baseline assessment and necropsy is consistent with their hypermetabolic state. A cause-effect relationship would be strengthened through investigation of alternative explanations, including the potential that neonatal SSRI exposure increases the stress/catabolic response to phenotypic assessment or the stress/hyperactivity response to isolation within the home cage. The SSRI-induced hypermetabolic state (increased resting expenditure) is itself a novel finding with a number of potential etiologies. Notably, the lean body habitus induced by neonatal SSRI exposure may both account for the increase in metabolic rate (as indexed by body weight) and be a manifestation of the hypermetabolic state. Among factors known to increase metabolic rate, activation of the adrenosympathetic or thyroid axes merit further consideration, especially in light of observational studies that have found an association between intrauterine SSRI exposure and postnatal hypothalamic alterations [13].

To further explore the molecular etiology for the persistent hypermetabolic state, we assessed cortical and midbrain TPH expression. SSRI-exposed male mice had a dramatic increase in expression of TPH2. While statistical analysis only reached significance in the cortex, similar effects were apparent in the midbrain. Consistent with studies in humans [42], we detected lower TPH2 expression in the cerebral cortex then in the midbrain section containing the dorsal raphe nucleus. Although there is currently no evidence this cortical expression substantially contributes to central serotonin synthesis, increased cortical TPH expression and activity has been associated with suicide in humans and altered stress hormone levels in rats [43, 44]. Further studies with region-specific TPH2 overexpression or inactivation will be needed to assess the effects of cortical TPH2 on central serotonin levels and behavioral phenotypes. Based on studies showing dose-dependent inhibition of TPH by SSRIs, it is also possible higher or more frequent exposures would also have greater effects on adult TPH expression and metabolic parameters.

Our results in male mice are in stark contrast with the reduced TPH immunoreactivity seen in the rat dorsal raphe nucleus following citalopram administration from P8–P21. Potential explanations for the discrepancy include: differences in mRNA versus protein expression (e.g., presence of RNA interference or instability), SSRI-specific or species-specific effects, and vastly different windows of exposure. Our data showing increased TPH2 after sertraline exposure is consistent with the increase in TPH mRNA and protein expression seen during sertraline or fluoxetine administration to rats [45]. Although parallel changes in mRNA and protein have been reported following sertraline exposure, it is possible that TPH1 and TPH2 are regulated at both transcriptional and translational levels. Recently developed TPH1 and TPH2 antibodies may permit analysis of isoform-specific expression with future cohorts of mice [46]. Interestingly, during acute citalopram administration, there is a decrease in TPH2 mRNA that does not translate to a change in protein levels [47], highlighting the importance of SSRI-specific research.

In our investigations, there was a clear impact of sex and a lesser effect of ovarian status on SSRI-induced TPH2 expression, suggesting the programmed alterations in TPH2 expression were likely established before pubertal development. Notably, we previously demonstrated male mice have a delayed neonatal window of developmental susceptibility [27], consistent with the female advantage seen following premature delivery [48]. It is possible an earlier window of exposure would be necessary to induce similar programming effects in females.

Our study is one of the first investigations to report long-term outcomes in SSRI-exposed female as well as male mice. Other than TPH2 mRNA expression and adult brain weights, very similar phenotypes were seen in male and female mice, in contrast to the sex-specific differences often reported in undernutrition or reduced uterine perfusion models of metabolic and cardiovascular programming [27, 49, 50]. Because the gene expression studies were not completed at the same age in male and female mice and the male mice did not undergo survival surgery, direct comparison of male and female TPH expression patterns are difficult. Regardless, the lack of correlation between central TPH2 expression and the hypermetabolic aspect of the post-SSRI syndrome led us to explore alternative etiologies.

Prior studies suggested TPH1 is expressed in the cortex during neurodevelopment and we hypothesized SSRI exposure might trigger a persistence of this immature expression pattern [51]. While ours confirmed an increased expression in the cortex and pineal sample relative to that in the midbrain, there was no significant programming effect seen. Further studies are needed focused on pineal gland mRNA and protein expression of enzymes within the melatonin synthetic pathway.

We were interested in melatonin for a number of reasons. Melatonin and serotonin share metabolic pathways including tryptophan hydroxylase conversion of tryptophan to the common precursor, 5-hydroxytryptophan. Exogenous melatonin has demonstrated efficacy in animal models of obesity with weight loss attributed to an increase in metabolic rate, potentially through noradrenaline sensitization or increased mitochondrial respiration [52–54]. The increased melatonin sulfate excretion seen in female mice following neonatal SSRI exposure may thus contribute to the associated hypermetabolic state. Because caloric intake, metabolic rate and TPH1 expression did not show sex-specific differences, additional male mice were not generated for the determination of urinary melatonin excretion. Notably, TPH is not the rate-limiting enzyme in melatonin production. Further studies, including microdialysis, are needed to define the circadian pattern of serotonin and melatonin production within discrete brain regions. Likewise, serotonin receptor antagonists and/or pinealectomy would help disentangle the importance of these signaling pathways in programmed mice.

In conclusion, the exposure of neonatal mice to the most commonly prescribed SSRI led to a post-SSRI syndrome associated with a hypermetabolic state, an upregulation of TPH expression in male mice, and increased melatonin excretion in female mice. The anorexigenic effects of serotonin and melatonin, as well as the lack of obesity in male and female mice, suggest the hyperphagia is not a primary phenotype, and it may be a compensation for the increased metabolic rate. In addition to translational mechanistic studies, clinical assessments are critically needed to assess the metabolic and neuroendocrine status of infants inadvertently exposed to maternal SSRI therapy.

## Figures and Tables

**Table 1:** From postnatal day 1 to 14, mice received injections of saline (control) or the SSRI sertraline. After obtaining adult phenotypes, tissue weights were obtained from 11- to 12-month-old female and 6- to 9-month-old male mice. *P < 0.05 versus control by Student's *t*-test

		Female	Female	Male	Male
		Control	SSRI	Control	SSRI

*N*		15	14	8	10
Body Weight	(g)	35.3 ± 1.6	29.6 ± 1.7 *	39.7 ± 2.1	35.0 ± 1.0*
Brain	(mg)	457 ± 4	458 ± 4	421 ± 12	406 ± 8
	(mg/g)	13.3 ± 0.6	16.1 ± 0.9 *	10.9 ± 0.7	11.3 ± 0.4
Liver	(mg)	1251 ± 103	1241 ± 70		
	(mg/g)	37 ± 3	42 ± 2		
White adipose	(mg)	2688 ± 308	1761 ± 396		
	(mg/g)	73 ± 6	53 ± 9		
Brown adipose	(mg)	127 ± 12	109 ± 12		
	(mg/g)	3.6 ± 0.3	3.6 ± 0.3		
